# 3-nitropyridine analogues as novel microtubule-targeting agents

**DOI:** 10.1371/journal.pone.0307153

**Published:** 2024-11-07

**Authors:** Jean Herman, Els Vanstreels, Dorothée Bardiot, Andrea E. Prota, Natacha Gaillard, Ling-Jie Gao, Thomas Vercruysse, Leentje Persoons, Tinne Daems, Mark Waer, Piet Herdewijn, Thierry Louat, Michel O. Steinmetz, Steven De Jonghe, Ben Sprangers, Dirk Daelemans

**Affiliations:** 1 4AZA Biosciences, Leuven, Belgium; 2 Interface Valorisation Platform (IVAP), KU Leuven, Leuven, Belgium; 3 Laboratory of Molecular Immunology, Department of Microbiology, Immunology and Transplantation, Rega Institute, KU Leuven, Leuven, Belgium; 4 Department of Pediatric Nephrology and Solid Organ Transplantation, University Hospitals Leuven, Leuven, Belgium; 5 Molecular Genetics and Therapeutics in Virology and Oncology Research Group, Department of Microbiology, Immunology and Transplantation, Rega Institute, KU Leuven, Leuven, Belgium; 6 Laboratory of Biomolecular Research, Center for Life Sciences, Paul Scherrer Institut, Würenlingen, Switzerland; 7 Laboratory of Medicinal Chemistry, Department of Pharmaceutical and Pharmacological Sciences, Rega Institute, KU Leuven, Leuven, Belgium; 8 University of Basel, Basel, Switzerland; 9 Molecular Structural and Translational Virology Research Group, Department of Microbiology, Immunology and Transplantation, Rega Institute, KU Leuven, Leuven, Belgium; 10 Department of Nephrology, University Hospitals Leuven, Leuven, Belgium; Lahore University of Management Sciences, Syed Babar Ali School of Science and Engineering, PAKISTAN

## Abstract

Microtubule-targeting agents are an important class of anti-cancer drugs; their full potential is however not realized because of significant myelotoxicity and neurotoxicity. We here report 3-nitropyridine analogues as a novel group of microtubule-targeting agents with potent anti-cancer effects against a broad range of cancer types. We show that these 3-nitropyridines induce cell cycle arrest in the G2-M phase and inhibit tubulin polymerization by interacting with tubulin. Determination of the tubulin–4AZA2996 structure by X-ray crystallography demonstrated that this class of compounds binds to the colchicine-site of tubulin. Furthermore, the anti-cancer effect was demonstrated both *in vitro* and *in vivo* in a murine heterotopic xenograft model of colon cancer. When administered intravenously, 4AZA2891 effectively inhibited cancer growth. Whereas 3-nitropyridine compounds do not induce myelotoxicity at pharmacological doses, the neurotoxicity associated with microtubule-targeting agents is still present.

## Introduction

Microtubules play a critical role in intracellular transport, protein trafficking, and cell division, and are thus an attractive target for anti-cancer therapy. Microtubules are composed of αβ-tubulin heterodimers which associate head-to-tail to form protofilaments and then laterally to form a tube. Microtubule-targeting agents alter the stability and dynamics of tubulin association and dissociation to and from microtubule ends, respectively. Available microtubule-targeting agents are central in the treatment of a wide variety of adult and pediatric cancers [1, 2]. Modulators of microtubule dynamics are broadly categorized in microtubule stabilizing (e.g. taxanes, epothilones) and destabilizing (e.g. vinca alkaloids, colchicine) agents [3] and can be further classified based on their binding site on tubulin [4]. Ultimately, all microtubule-targeting agents promote cancer cell death by arresting mitosis resulting in a continued activation of the spindle assembly checkpoint and apoptosis [5, 6].

A major drawback of currently available microtubule-targeting agents is their toxicity, most importantly hematologic and neurologic side effects. These side effects often require dose reduction or discontinuation of these drugs [7, 8]. Peripheral neuropathy caused by microtubule-targeting agents is caused by microtubule damage in the long axons of peripheral nerves [9], and results in partially or non-reversible symptoms [10, 11]. Ideally, a microtubule-targeting agent would efficiently induce mitotic arrest and apoptosis in rapidly dividing cancer cells without significantly affecting non-dividing cells. Therefore, continued research to develop microtubule-targeting agents with a better safety profile is warranted.

In order to identify new anticancer agents, a corporate and proprietary compound library was screened in a cytotoxicity assay, leading to the identification of 2-morpholinoethylamino-3-anilino-3-nitropyridines as very potent cytotoxic agents. In this manuscript, the *in vitro* and *in vivo* biological profiling, target identification and X-ray crystallography data are reported.

## Material and methods

### Synthesis of 4AZA2891 and 4AZA2996

Detailed methods for the synthesis and structure determination of these new chemical entities are provided in the S1 File.

### Cells and cell lines

All cancer cell lines (HT-29: colorectal adenocarcinoma, HCT-116: colorectal carcinoma, A549: lung epithelial carcinoma, PC3: prostate adenocarcinoma, Jurkat: acute T-cell leukemia, BC-1: primary effusion lymphoma) and the normal human embryonal lung fibroblast cell line MRC-5 were acquired from the American Type Culture Collection (ATCC). All cell lines were cultured as recommended by the suppliers. Culture media were purchased from Gibco (Thermo Fisher Scientific) and supplemented with 10 to 20% fetal bovine serum (HyClone, GE Healthcare Life Sciences) and 20–25 μg/mL gentamicin (Gibco, Thermo Fisher Scientific). All cells lines were thawed upon receipt from the distributor, amplified and subsequently batches were frozen in liquid nitrogen. Thawed lots were kept in culture no longer than 6 months.

Buffy coat preparations from healthy donors were obtained from the Blood Transfusion Center in Leuven, Belgium. Peripheral blood mononuclear cells (PBMC) were isolated by density gradient centrifugation over Lymphoprep™ (Axis Shield PoC AS; density 1.077 ± 0.001 g/mL) and cultured in cell culture medium (DMEM/F12, Gibco Thermo Fisher Scientific) containing 8% fetal bovine serum. Each donor consented to the use of his blood for research purposes.

Reference inhibitor compounds paclitaxel, vincristine and vinblastine were obtained from Selleckchem (Houston, Texas, USA) and stock solutions were prepared in DMSO at concentrations as specified in the manuscript.

### Clonogenic assays

All experiments were performed under standard cell culture conditions at 37°C and 5% CO_2_. Colorectal adenocarcinoma HT-29 cells were seeded at 300 cells/well in triplicate in six well microtiter plates in RPMI-1640 medium supplemented with 10% fetal bovine serum and 25 μg/ml gentamicin (complete medium). Twenty-four hours later, the compounds were added in complete medium in final concentrations ranging from 10 μM to 0.1 nM. Cells were allowed to grow until they formed colonies for 6 days. The colonies were fixed with 100% methanol, stained with Trypan blue 0.4% in saline and the number of colonies was counted. The number of colonies in the treated wells was normalized to the number obtained in the untreated wells. Three biological independent experiments were performed for each compound.

### Cell viability, cell cycle and apoptosis

Cell viability in cancer cell lines treated with compound was measured in a 3-day MTS cell proliferation assay using the CellTiter 96 AQ_ueous_ Non-Radioactive Cell Proliferation Assay reagent (Promega) according to the manufacturer’s instructions. Absorbance was read at 490 nm on a SaFire II microplate reader (Tecan). CC50 values were calculated by fitting the obtained MTS cell viability data in GraphPad Prism using a 4-parameter log–based fit for inhibitors. Apoptosis induction and cell death upon compound treatment in PBMC was measured using the Alexa Fluor 488 Annexin V/Dead Cell Apoptosis Kit (Thermo Fisher Scientific), with readout on a FACSCanto II flow cytometer (BD Biosciences). For cell cycle analysis, Jurkat cells were seeded at 400,000 cells per well in 12-well tissue culture plates and treated with the test compounds at the indicated concentrations for 24 h. Cells were then harvested by centrifugation at 400 x g, washed in PBS and transferred to an 8-well μ-Slide (Ibidi) that had been pretreated with 0.1% (w/v) poly-L-lysine (Sigma). Cells were allowed to adhere to the slides, fixed with 4% PFA in PBS for 10 minutes, washed and stained with DAPI. Plates were imaged on a CX5 High Content Screening device (ThermoFisher Scientific) employing the Cell Cycle Analysis bio-application, analyzing a minimum of 3000 cells per condition. The compounds were evaluated *in duplo* in two independent experiments.

### NCI60 anticancer drug screen

Compounds 4AZA2891 and 4AZA2996 were tested in the Nationcal Cancer Institute (NCI) 60 panel of 60 human tumor cell lines representing leukemia, melanoma and cancers of the lung, colon, brain, ovary, breast, prostate, and kidney. The screening methodology used in the NCI60 Cell 5-Doses screen is described in detail at https://dtp.cancer.gov/discovery_development/nci-60/methodology.htm. Briefly, cells are seeded in 96 well plates at an appropriate density and incubated for 1 day. After 1 day, some of the plates are processed to determine a time zero density. To the remaining plates, compounds are added over a 5-log M concentration range (1 log dilutions from 10^-4^M to 10^-8^M). Plates are incubated for two additional days, then fixed and stained with sulforhodamine B. Growth inhibition is calculated relative to cells without drug treatment and the time zero control [12]. The GI_50_ is the concentration of a compound that causes 50% growth inhibition, relative to the no drug control.

### Fluorescence microscopy

Non-small cell lung carcinoma A549 cells were seeded at 50,000 cells/well in an 8-well Nunc™ Lab-Tek™ chambered coverglass slide. After overnight incubation, they were treated with compound or carrier (DMSO) for 4 hours and then fixed with 4% PFA, washed and permeabilized (0.2% Triton X-100 in PBS). Further treatment was performed according to standard immunofluorescence procedures, employing 10% normal goat serum (Thermo Fisher Scientific) as blocking medium and as antibody diluent. Employed antibodies were mouse anti-alpha tubulin (sc-5286, Santa Cruz Biotechnology) at 1:300 dilution and secondary goat anti-mouse IgG conjugated to Alexa Fluor® 488 (A11001, Invitrogen, Thermo Fisher Scientific). Cell nuclei were counterstained with DAPI. Images were collected with a Leica TCS SP5 confocal microscope employing a HCX PL APO 63x (NA 1.2) water immersion objective.

### Tubulin polymerization assay

Tubulin polymerization *in vitro* was analyzed using the fluorescence-based tubulin polymerization assay (Cytoskeleton, Inc.), according to the manufacturer’s guidelines. Fluorescence was read on a SaFire II microplate reader (Tecan).

### Crystallization, data collection, and structure determination

Crystals of the T_2_R-TTL complex (composed of two αβ-tubulin heterodimers, the stathmin-like protein RB3 protein and tubulin tyrosine ligase) were generated as described [13, 14]. Suitable T_2_R-TTL crystals were soaked over night in reservoir solutions (4% PEG 4K, 10% glycerol, 30 mM MgCl_2_, 30 mM CaCl_2_, 0.1 M MES/Imidazole, pH 6.7) containing 5 mM 4AZA2996 and subsequently flash cooled in liquid nitrogen following a brief sequential transfer into cryo solutions containing the reservoir supplemented with 16% and 20% glycerol. All data were collected at beamline X06DA at the Swiss Light Source (Paul Scherrer Institut, Villigen PSI, Switzerland). Images were indexed and processed using XDS [15]. Structure solution using the difference Fourier method and refinement were performed using PHENIX [16]. Model building was carried out iteratively using the Coot software [17]. Data collection and refinement statistics for the T_2_R-TTL-4AZA2996 complex are given in S1 Table. Molecular graphics and analyses were performed with PyMOL (The PyMOL Molecular Graphics System, Version 2.3.2, Schrödinger, LLC).

#### PDB ID codes

Coordinates and structure factors of the T_2_R-TTL-**4AZA2996** complex have been deposited at the Protein Data Bank (www.rcsb.org) under accession number PDB: 8R6O. Authors will release the atomic coordinates and experimental data upon article publication.

### Animals

Animals were housed at KU Leuven animal facilities with a 12-hour light/dark cycle; food and water were provided ad libitum. Animals were acclimatized for at least 7 days before experimentation. The studies were carried out in accordance with the Guide for the Care and Use of Laboratory Animals as adopted and promulgated by the U.S. National Institutes of Health and were approved by the Institution’s Animal Ethic Committee.

### Pharmacokinetics in rats

The pharmacokinetic profile of 4AZA2891 was investigated in male Sprague-Dawley rats after intravenous and oral administration of a dose of 2 mg/kg and 20 mg/kg, respectively. The rats were purchased from Charles River Laboratories (Belgium) and 3 rats were included in each dosing route. 4AZA2891 was formulated either as a solution at 1 mg/ml in glucose 5% with 2 equivalents of H_3_PO_4_ for the IV dosing or as a solution at 4 mg/ml in water with 2 equivalents of H_3_PO_4_ for the oral dosing. Both formulations were heated for 1 hour at 80°C in order to obtain a clear solution. Serial venous blood samples, taken by eye puncture under isoflurane inhalation anesthesia, were collected in heparinized tubes. The plasma fraction was immediately separated by centrifugation 2 min at 13,000 g and stored at -80°C until analysis. Plasma concentrations were measured using liquid chromatography/mass spectrometry/mass spectrometry (LC/MS/MS).

### Pharmacokinetics and tissue distribution in mice

Female CD1 mice were purchased from Harlan (Harlan Netherlands BV) and 7–8 weeks old mice were injected intravenously (IV) with 20 mg/kg 4AZA2891 formulated at 2 mg/ml as described above. After dosing, the mice (n = 3 /time point) were exsanguinated by retro-orbital bleeding under anesthesia with avertin (2,2,2-tribromoethanol) given intraperitoneally and the organs were flushed in situ by intracardiac injection of 10 ml ice-cold saline, the vena cava inferior being cut to allow evacuation of the effluent. The plasma, obtained after centrifugation of the heparinized blood for 2 min at 13,000 g, and the harvested tissues, after homogenization with Ultra-Turrax, were stored at-80°C until analysis by LC-MS/MS.

### Bioanalysis of plasma and tissue samples

Every plasma and tissue sample was spiked with internal standard followed by the addition of 4 volumes of methanol. The samples were kept for 30 min on ice prior to centrifugation 10 min at 12,000 RPM in order to remove precipitated proteins. The supernatant was analyzed for the presence of 4AZA2891 using LC/MS/MS (Finnigan LCQ advantage Max (ion trap) mass spectrometer with Surveyor HPLC System (Thermo Finnigan)).

As standard for the bioanalysis, 4AZA2891 was diluted stepwise. Each dilution was added to blank control plasma or to blank control tissue homogenate additionally spiked with internal standard, giving rise to standard curves from 5 to 5,000 ng/ml. The lower limit of quantification was 5 to 10 ng/ml depending on the tissues.

Non-compartmental pharmacokinetic analysis was performed on the plasma concentration-time data. PK Solutions 2.0 software (Summit Research Services) was used to calculate the PK parameters.

### HT-29 xenograft tumor model

Seven-week-old NMRI nude male mice were obtained from Janvier Labs (France) and injected with 10^7^ HT-29 cells intradermally on the flank. When tumors reached approximately 100 mm^3^, mice were distributed into three groups of four to six mice to give the same average tumor size per group and randomly assigned a treatment condition. Mice were injected IV with 4AZA2891 at 15 mg/kg following a Q1D5x2W administration schedule or at 30 mg/kg following a Q2D3x2W administration schedule or with the vehicle (Q1D5x2W). 4AZA2891 was formulated before each injection at 3 mg/ml in 5% glucose with 2 equivalent H_3_PO_4_ and warmed at 80°C for 1 h to obtain a clear solution. Tumor volume was measured as (width^2^ × length)/2 in mm^3^ and weight gain/loss and survival were monitored. T/C ratio was calculated as the ratio of the relative tumor volume average. The health status and behaviour of the animals were assessed daily for well-being. At the end of the experiment (day 35 after treatment intiation)) or in the events of signs of distress, cachexia or excessive tumor growth, the mice were humanely sacrificed by CO_2_ inhalation followed by cervical dislocation.

### Myelotoxicity in mice

Female Balb/c mice from Janvier Labs, 8–10 weeks old, were injected IV daily for 6 days with 4AZA2891 at 15 mg/kg or with the vehicle (4 mice per treatment group). The compound was formulated at 0.75 mg/ml in glucose 5% with 2 equivalents H_3_PO_4_ as described above. Mice were daily weighted and assessed clinically. On day 7, mice were exsanguinated by retro-orbital bleeding under anesthesia with avertin (given intraperitoneally); blood was collected in EDTA tubes and was subjected to an automated blood count (Sysmex XE 2100 Hematology Analyser, Sysmex Europe GmbH). Femurs were fixed in formaldehyde, decalcified, and paraffin embedded. Sections (4.5 μm) were stained with hematoxylin/eosin (H&E) for histopathological analysis at Novaxia (France).

### Neurite outgrowth

Neurite outgrowth assay was performed by Fluofarma (Pessac, France). Primary cortical neurons were harvested from E19 Sprague-Dawley (Janvier Labs) rat embryos, dissociated and plated at 10,000 per well in a tissue-culture treated 96 well plate, in neuronal medium (SM2 matrix independent growth medium, Stem Cell Technologies). Neurons were followed by phase contrast imaging with 1 image acquired every 4 hours, using an Incucyte Zoom (Essen BioScience) with a 20x objective. Four fields per well were acquired. After 24 hours, cells were treated with test compounds and neurite outgrowth was followed for 72 hours post-treatment. All measures were performed in triplicate in one experiment.

Neurite outgrowth kinetics were analyzed from each phase contrast image using the Neurotrack module of the Incucyte Zoom platform. Neurite length and branch points were obtained for each time point. The area under the curve (AUC) was calculated in each experimental condition (GraphPad Prism 6, GraphPad Software).

### Statistical analyses

Data are expressed as mean ± standard deviation (SD) where applicable and unless otherwise specified. The mean difference between the control and treated groups was analyzed by Student’s t-test for the HT29 xenograft tumor model using GraphPad Prism 6 (San Diego, USA). A p-value ≤0.05 was considered statistically significant.

## Results

### Inhibition of the growth of cancer cell lines

**Fig 1:**
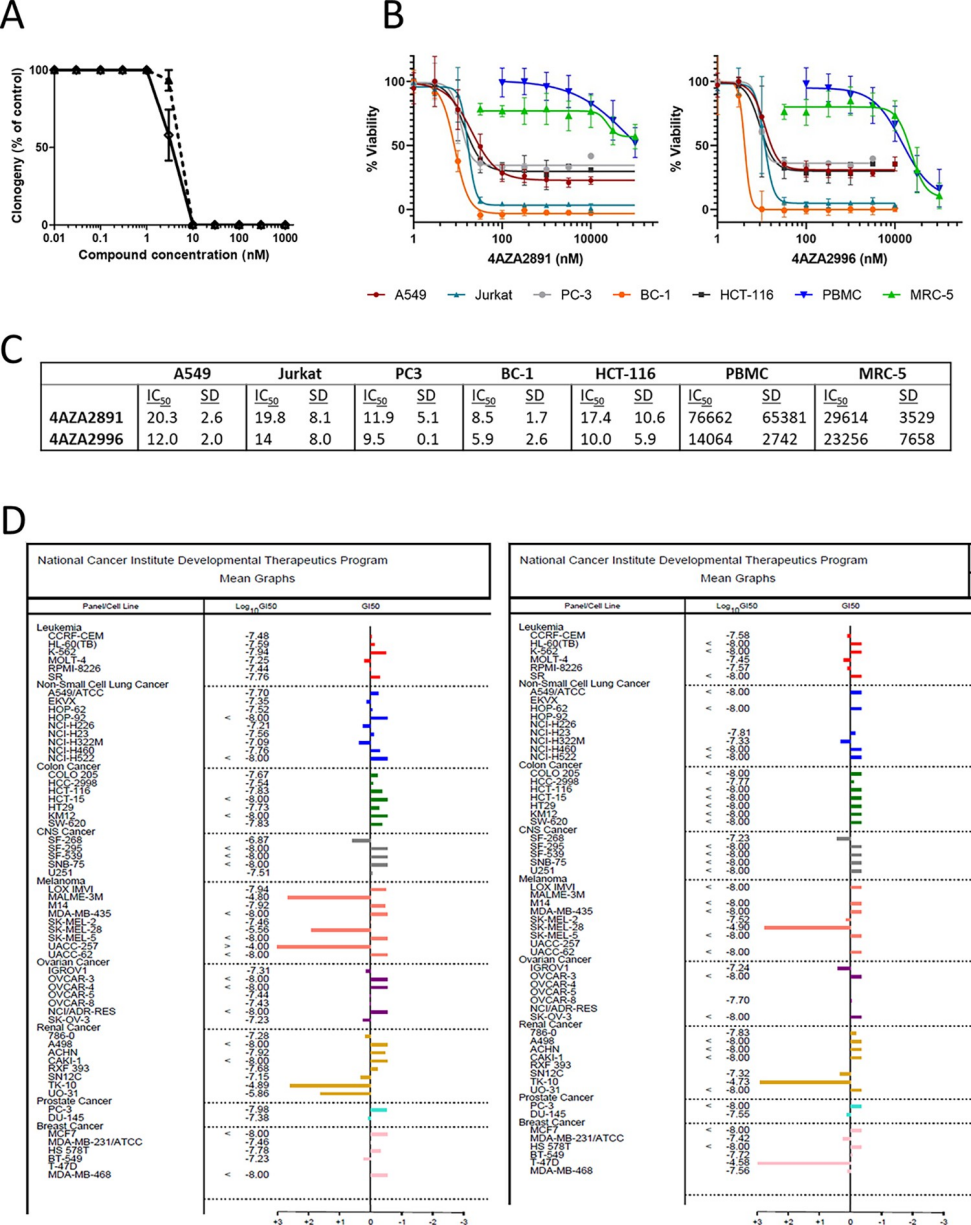
Inhibition of the growth of cancer cell lines. (**A**) Survival of HT-29 cells exposed to increasing concentrations of 4AZA2891 (dashed line) and 4AZA2996 (solid line) in a clonogenic assay. HT-29 cells were seeded and compounds were added 24 hours later. After 6 days of incubation, cells were fixed with methanol and stained with Trypan blue. Colonies were counted and numbers were normalized to the number of colonies in the non-treated wells. The results are expressed as mean ± SEM of three independent experiments. (**B**) 4AZA2891 and 4AZA2996 induce cytotoxicity in hematological and solid cancer cell lines while sparing normal cells. Cells were treated with different concentrations of compound, and 72 hours later viability was measured by MTS assay or in the case of PBMC by annexin V/PI flow cytometry. Datapoints are mean ± SD (n = 2–4). (**C**) IC50 values (nM) as calculated from the linear portions of the log dose response curves depicted in Fig 1B. Numbers represent average values of two to four experiments ± S.D. (**D**) 4AZA2891 (left) and 4AZA2996 (right) were assessed in the NIH panel of cancer cell lines at 5 concentrations and the GI_50_ was determined for each cell line from the dose-response curves. The mean GI_50_ for each compound across all cell lines was calculated and is represented as a vertical line in the graphs. This mean is assigned a value of zero and all the Gi50’s of cell lines are plotted relative to it. The bars representing cell lines that require concentrations higher than the mean for inhibition point to the left; those representing cell lines that are more sensitive to the compound point to the right. The mean GI_50_ (log_10_) for 4AZA2891 is -7.45 molar and for 4AZA2996–7.66 molar.

We screened compounds from a corporate and proprietary compound library for their anti-proliferative potential by a clonogenic assay on the human colorectal adenocarcinoma HT-29 cell line and identified two 3-nitropyridine compounds as strong inhibitors of HT-29 cell proliferation with an IC_50_ below 10 nM. Compound 4AZA2891 has an IC_50_ of 5.4 nM, whereas 4AZA2996 displays an IC_50_ value of 4.0 nM (Fig 1A).

Next, we investigated the anti-proliferative activity of these compounds on a selection of solid and hematological cancer cell lines and compared this to their effect on the viability of normal PBMC and normal lung fibroblast cells (MRC-5). While nanomolar concentrations of the 3-nitropyridine compounds cause strong cytotoxic effects in all cancer cell lines tested, we did not observe decreased viability in normal cell lines in this concentration range, suggesting that these compounds are selectively active against rapidly dividing cancer cells. (Fig 1B and 1C).

We subsequently evaluated 4AZA2891 and 4AZA2996 in the NCI-60 Human Tumor Cell Lines Screen, an established panel of cancer cell lines representing leukemia, melanoma and cancers of the lung, colon, brain, ovary, breast, prostate, and kidney. Both compounds demonstrate potent anti-proliferative activities against a wide range of cancer cell types. The mean GI_50_ across the panel is 35.5 nM for 4AZA2891 and 21.9 nM for 4AZA2996. The GI_50_ is lower than 10 nM in 17 out of the 59 cell lines tested for 4AZA2891 and in 31 of the 50 cell lines tested for 4AZA2996. Only a few cell lines are less sensitive to the compounds with an IG_50_ above 10^−6^ molar (Fig 1D).

Analysis of anti-proliferative effect on the panel of cancer cell lines indicates that 3-nitropyridine compounds are not substrates for the multidrug resistance efflux pumps, as P glycoprotein or MRP, since the compounds are still efficient on HCT-15 [18–20], NCI/ADR-res [19, 20], UO-31 [19–21] and CAKI-1 [19, 22].

### Characterization of the mechanism of action of the 3-nitropyridine compounds

**Fig 2:**
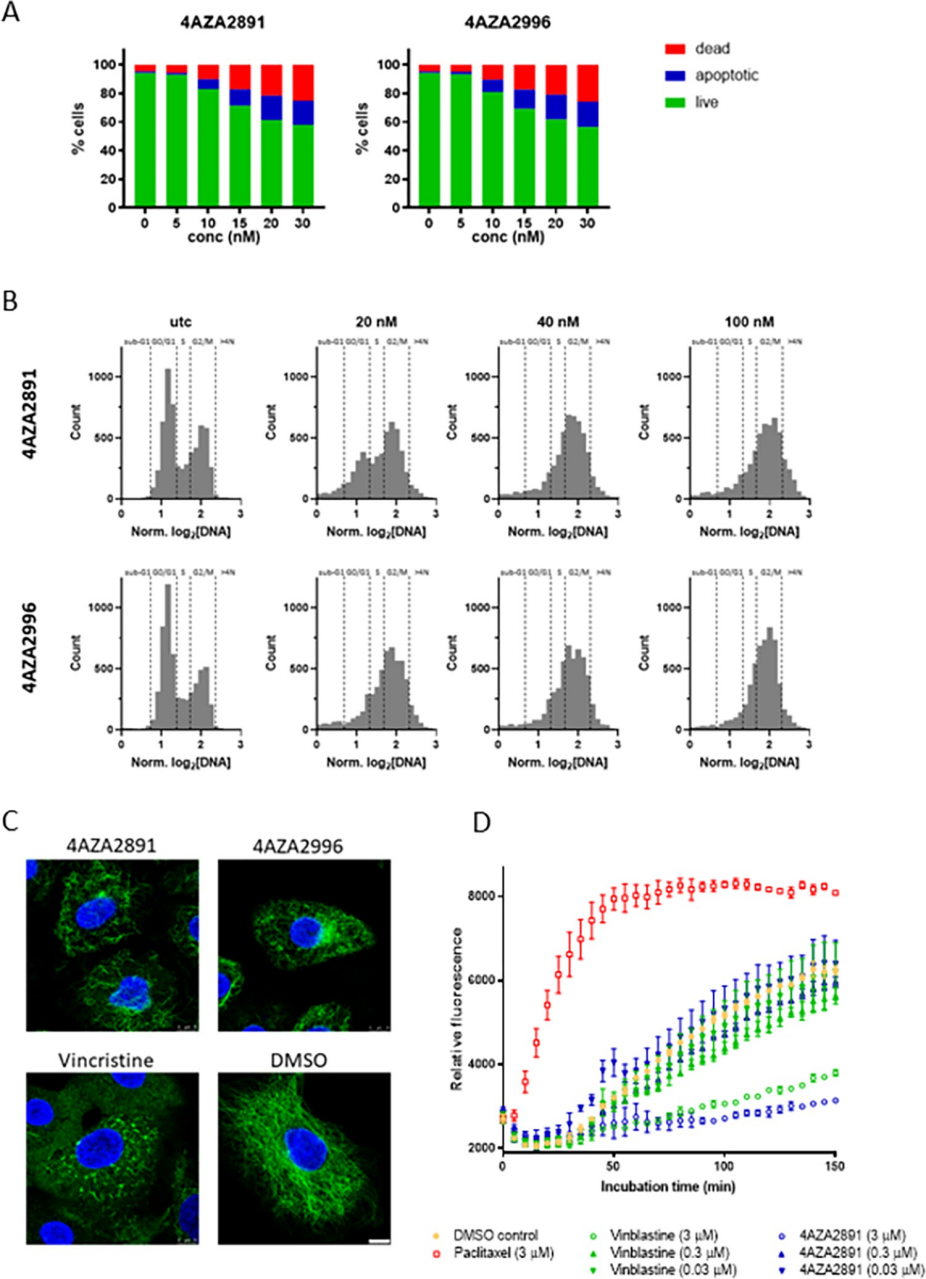
Mechanism of action of the 3-nitropyridine compounds. (**A**) 4AZA2891 and 4AZA2996 induce apoptosis in Jurkat cells. Apoptosis was measured by annexin V/PI staining. Cells were treated with different concentrations of compound or carrier (DMSO) and 24 hours later apoptotic and dead cells were stained and subsequently analyzed by flow cytometry. (**B**) 4AZA2891 and 4AZA2996 induce G2 cell cycle arrest in Jurkat cells. Cells were treated with different concentrations of compound or carrier (DMSO) for 24 hours and then stained with DAPI cell cycle profiling by high content imaging. Both compounds cause a clear reduction of the G0/G1 while the G2 as well as the subG1 population increases. (**C**) 4AZA2891 and 4AZA2996 disintegrate the microtubule network. Immunofluorescence staining of alpha-tubulin in A549 cells treated for 4 hours with 250 nM of compound 4AZA2891, 4AZA2996 or reference compound vincristine. Green: α-tubulin, blue: DAPI. Scale bar: 10 μm. (**D**) 4AZA2891 dose-dependently inhibits tubulin polymerization *in vitro*. The polymerization of purified tubulin is measured by the increase in fluorescence due to the incorporation of a fluorescent reporter into microtubules as polymerization proceeds. 4AZA2891 dose-dependently causes, as vinblastine, a marked decrease in final polymer formation as compared to the DMSO control and in contrast to the effect observed for tubulin stabilizing reference compound paclitaxel.

To further evaluate the mechanism of cancer cell death upon compound treatment, we measured apoptosis induction in acute T cell leukemia Jurkat cells. Both compounds rapidly induce apoptosis in a dose-dependent manner (Fig 2A). Next, we analyzed the effect of 4AZA2891 and 4AZA2996 on cell cycle progression in Jurkat cells and observed a marked arrest in G2 phase (Fig 2B).

The selective anti-proliferative effect on cancer cells, the induction of apoptosis and the predominant arrest of the cells in G2/M phase of the cell cycle strongly suggest that the 3-nitropyridine compounds are tubulin-targeting agents. We therefore investigated the effect of the compounds on the integrity of the tubulin network by immunofluorescence staining of α-tubulin. Because their morphology allows for easy microscopic observation, we choose A549 lung carcinoma cells for these experiments. The 3-nitropyridine compounds caused disintegration of the microtubule network, comparable with the effect of the vinca alkaloids and the known tubulin-destabilizing reference compound vincristine (Fig 2C). We then performed an *in vitro* tubulin polymerization assay, which confirmed the dose-dependent inhibitory effect of 4AZA2891 on tubulin polymerization (Fig 2D).

**Fig 3:**
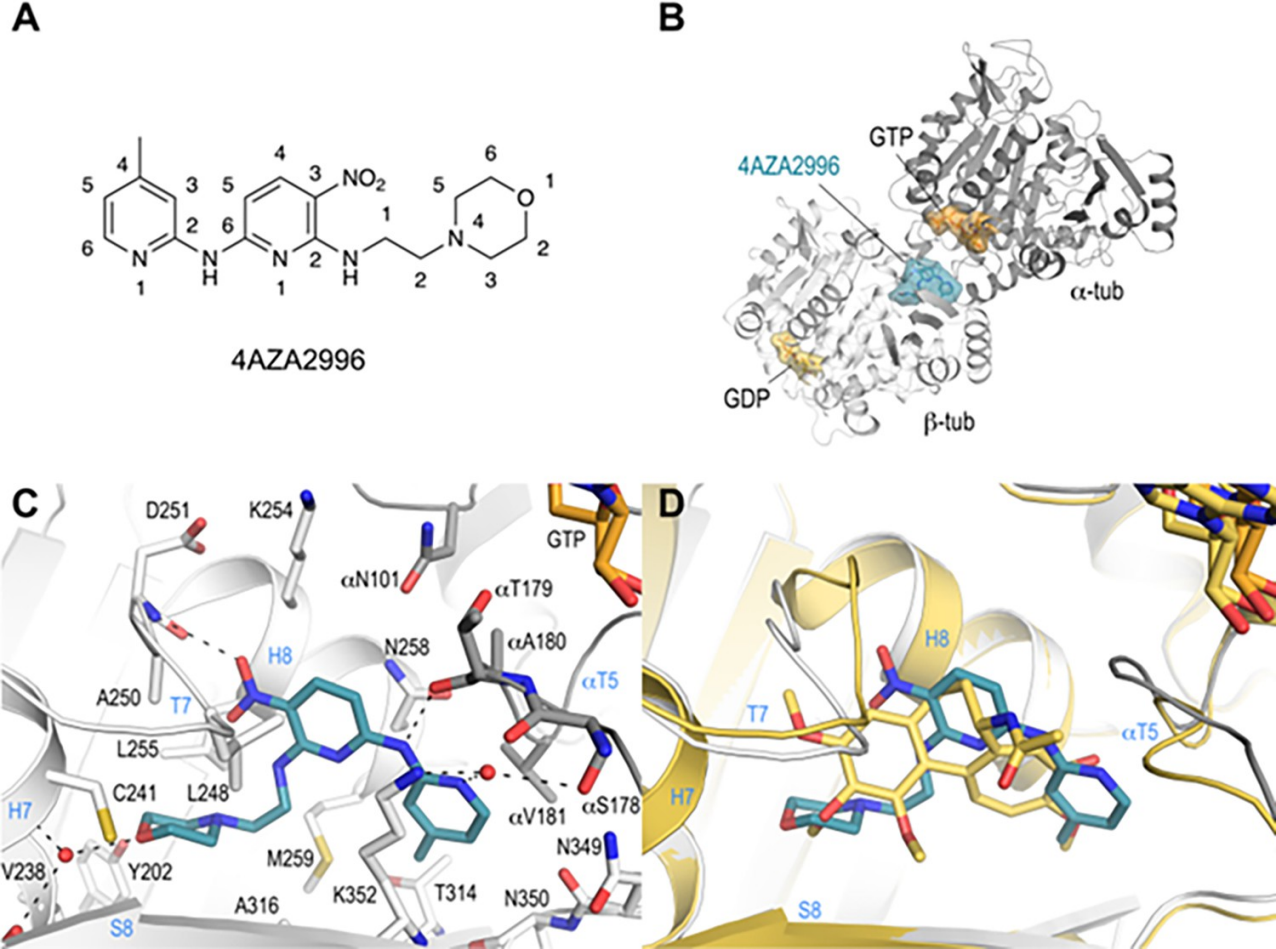
Co-crystallization of 4AZA2996 with tubulin. (**A**) Chemical structure of 4AZA2996 (*N*^6^-(4-methylpyridin-2-yl)-*N*^2^-(2-morpholinoethyl)-3-nitropyridine-2,6-diamine). (**B**) Ribbon representation of the tubulin-bound 4AZA2996 structure. The α- and β-tubulin chains are in dark and light gray, respectively. The ligand 4AZA2996 (teal) and the nucleotides (orange) are in sphere and stick representation, respectively. Oxygen and nitrogen atoms are colored in red and blue, respectively. (**C**) Close-up view of the atomic interaction network observed between 4AZA2996 (teal) and tubulin (gray). Interacting residues of tubulin within 4Å distance are shown in stick representation and are labeled. Oxygen and nitrogen atoms are colored in red and blue, respectively. Hydrogen bonds are depicted as black dashed lines. Secondary structural elements of tubulin are labeled in blue. For simplicity, only α-tubulin residues and secondary structural elements are indicated with an “α”. (**D**) Superposition of 4AZA2996 (teal) with colchicine (yellow-orange). The structures were superimposed onto the β-tubulin chains B of their respective T_2_R-TTL complexes. Only the ligands are shown in stick representation. Oxygen and nitrogen atoms are colored red and blue, respectively. Secondary structural elements of tubulin are labeled in blue.

To determine the tubulin-binding mode of the 3-nitropyridines, we soaked compound 4AZA2996 (Fig 3A) into a T_2_R-TTL crystal and determined the T_2_R-TTL−4AZA2996 complex structure by X-ray crystallography to 2.2 Å resolution (Fig 3; S1 Table; S2 Fig). 4AZA2996 binds to the colchicine-site of tubulin [23] at the intradimer interface (Fig 3B) and interacts with residues from strands βS8 and βS9 and helix βH8 of β-tubulin, and from the loop αT5 of α-tubulin (Fig 3C). The 2-morpholinoethylamino moiety of the ligand is anchored into a hydrophobic pocket in the β-tubulin subunit formed by residues βTyr202, βVal238, βCys241, βLeu242, βLeu255, βMet259, βAla316 and βIle318. The oxygen of the morpholino moiety establishes a water mediated interaction to both the main chain carbonyl and amine groups of βGly237 and βCys241 (Fig 3C). Additional hydrogen bonds are formed between the oxygen of the nitryl moiety and the backbone amide of βAsp251, between the *N*^*6*^-amine and the main chain carbonyl of αThr179 and between the amine of the 4-methylpyridine moiety and the side chains of βLys352 and of αSer178, respectively (Fig 3C).

A comparison of the binding mode of 4AZA2996 with that of colchicine (RMSD_chainB_ 0.53 Å over 423 C_α_-atoms (PDB ID 4O2B)); Fig 3D) reveals that both compounds occupy a similar space in the colchicine site, suggesting that 4AZA2996, like colchicine and other colchicine-site ligands (reviewed in [4]), prevents the compaction of the site, thereby inhibiting the “curved-to-straight” tubulin conformational transition occurring upon polymerization into microtubules [1, 2, 23].

### Pharmacokinetics of 4AZA2891 in Sprague Dawley rats

**Fig 4:**
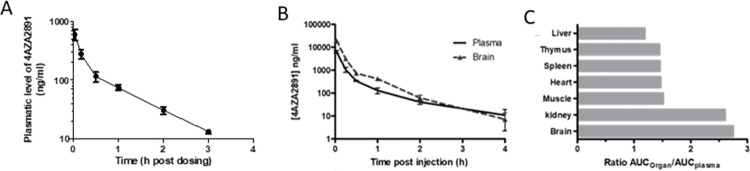
Pharmakokinetics and tissue distribution of 4AZA2891. (**A**) Sprague-Dawley rats (n = 3) were injected IV with 4AZA2891 at 2 mg/kg and blood was collected at the indicated time points. Plasma concentrations were measured using LC/MS/MS (lower limit of quantification of the bioanalysis: 10 ng/ml). (**B, C**) CD1 mice were injected IV with 4AZA2891 at 20 mg/kg and sacrificed at different time point post injection (3 mice per time point). (**B**) Concentration versus time curves of 4AZA2891 in plasma and brain. (**C**) Ratios of respective AUCs of organs over plasma.

In a next set of experiments, we investigated the pharmacokinetic profile and the oral bioavailability of 4AZA2891 in Sprague-Dawley rats after one IV dose of 2 mg/kg and an oral dose of 20 mg/kg. After IV administration, 4AZA2891 was detected in the plasma up to 3 hours (lower limit of quantification of the bioanalysis: 10 ng/ml) (Fig 4A). The area under the concentration–time curve (AUC_last_) in plasma was 253 ng.h/ml. The elimination half-life, volume of distribution at steady state, and systemic clearance were 0.8 hours, 7.4 l/kg, and 7.9 l/h per kg, respectively. Following an oral dose of 4AZA2891 at 20 mg/kg, plasma levels of 4AZA2891 were below the limit of quantification of the method at all time points, demonstrating that 4AZA2891 is not orally bioavailable.

### Pharmacokinetic and tissue distribution of 4AZA2891 in CD1 mice

Upon administration of 4AZA2891 at 20 mg/kg by IV bolus injection, its concentrations were measured in plasma and different organs (Fig 4B). The plasmatic pharmacokinetic parameters revealed an elimination half-life of 0.7 hours, a volume of distribution at steady state of 5.7 l/kg, and a systemic clearance of 15.4 l/h per kg. The area under the concentration–time curve (AUC_last_) in plasma was 1,292 ng.h/ml. Tissue distribution of 4AZA2891 revealed that it distributes readily to all tissues analyzed including the brain. The elimination half-lives in the different tissues range from 0.3 to 0.8 hours. Fig 4C shows the ratios of the tissues AUC over the plasma AUC. Interestingly, in brain and kidney, AUCs are 2.7 and 2.6 higher than in the plasma, suggesting that the compound goes preferentially to these tissues.

### Inhibition of tumor growth in a colorectal xenograft model *in vivo*

**Fig 5:**
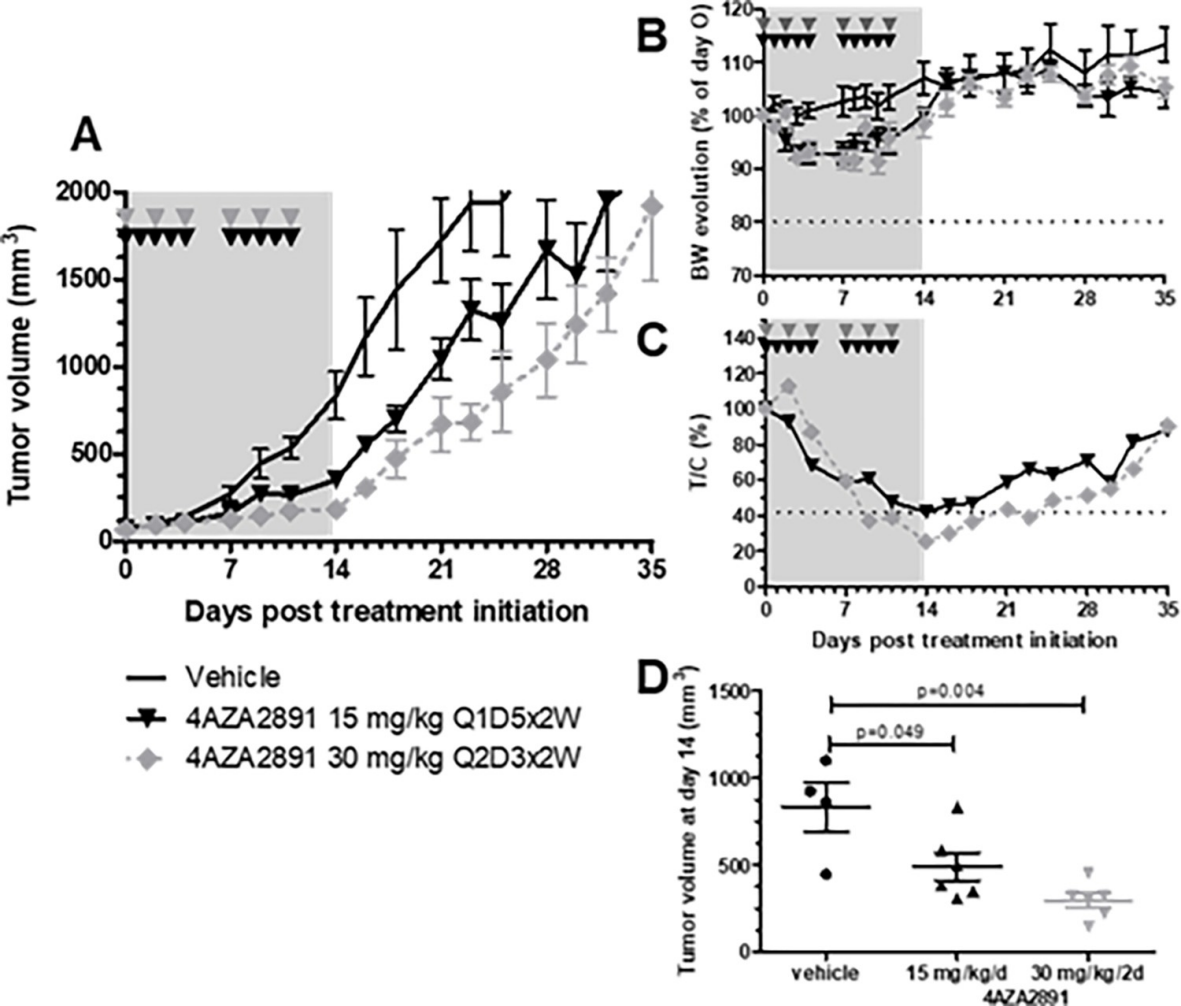
Antitumor effect of 4AZA2891 in a HT-29 xenograft model. (**A**) The efficacy of 4AZA2891 was evaluated in a xenograft model. 10^7^ HT-29 cells were injected subcutaneously in the flank of male nude mice. After tumor development, the mice were assigned to different treatment groups: vehicle, 4AZA2891 at 15 mg/kg/d administered 5 days a week for 2 weeks or 30 mg/kg/d every other day for 2 weeks. Treatments were given by IV administration. (**B**) Body weight evolution of mice for each treatment group. (**C**) Ratio treated over control relative tumor volume (T/C). (**D**) Tumor volume at the end of the treatment period. (The grey zones represent the treatment period; the arrowheads indicate the schedule of treatment administration).

We subsequently examined the therapeutic efficacy of 4AZA2891 in a mouse xenograft model of colon cancer (HT-29). Tumor bearing mice were treated with 4AZA2891 (15 mg/kg with a Q1D5x2W schedule or 30 mg/kg at Q2D3x2W) or vehicle administered IV. 4AZA2891 induced at both dosing regimen significant tumor growth inhibition (Fig 5). The 30 mg/kg dosing regimen showed greater efficacy than the 15 mg/kg regimen. At the end of the treatment period, the ratio of relative tumor volume of treated mice over control mice (T/C) reached 42% in the 15 mg/kg regimen and 34% in the 30 mg/kg regimen (Fig 5C). Both dosing regimens were well tolerated as reflected by the body weight evolution (Fig 5B). After cessation of treatment, tumor growth resumed at a rate almost similar to that of the vehicle controls (Fig 5A).

### Evaluation of 3-nitropyridine compound-induced myelotoxicity and neurotoxicity

**Fig 6:**
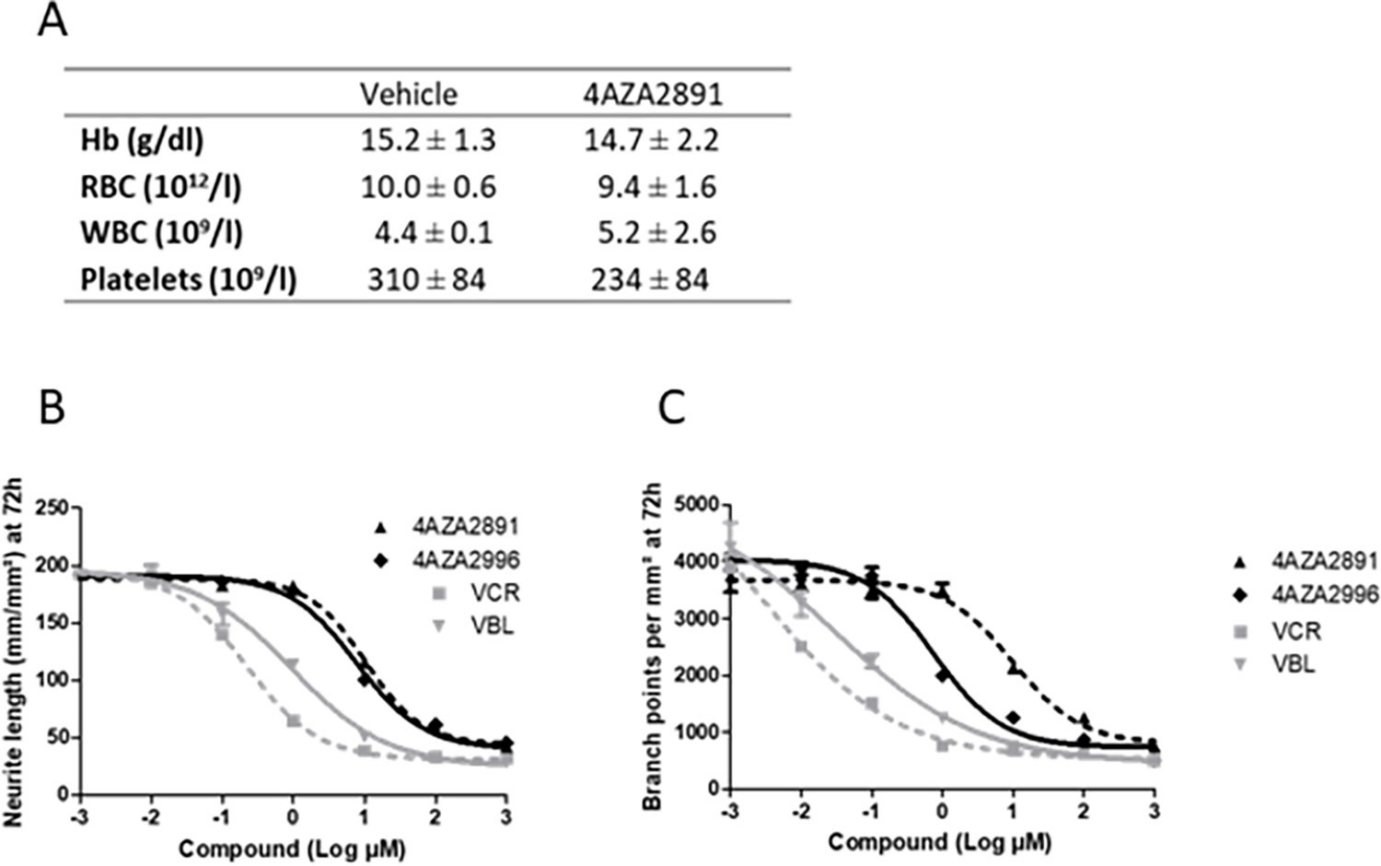
Myelotoxicity and neurotoxicity of 4AZA compounds. (**A**) Balb/c mice were treated IV once daily for 6 days with 4AZA2891 at 15 mg/kg or with the vehicle (4 mice per treatment group). On day 7, blood was collected and subjected to an automated blood count (Sysmex XE 2100 Hematology Analyser). Hb: hemoglobine; RBC: red blood cell; WBC: white blood cell. Data are mean ± SD. (**B, C**) *In vitro* neurotoxicity of 4AZA compounds, Vincristine (VCR) and Vinblastine (VBL). Primary cortical neurons were cultivated in presence of increasing doses of compounds. (B) Neurite length and (C) branch points were quantified at 72 hours. The measurements were performed in triplicate in one experiment.

As the major drawbacks of microtubule-targeting agents are myelo- and neurotoxicity, we next examined our compounds for these effects. Daily treatment of Balb/c mice with 4AZA2891 during 6 days at 15 mg/kg/d IV was well tolerated and did not result in significant body weight change, anemia, leukocytopenia or thrombocytopenia (Fig 6A). Histologic examination of the bone marrow was normal.

To assess neurotoxicity, we examined the effect of 4AZA2891 and 4AZA2996 on cortical neurons in the neurite outgrowth assay, an established *in vitro* assay. Both vincristine and vinblastine were tested as controls. All compounds significantly inhibited the neurite development at low concentrations (Fig 6B and 6C). The IC_50_ for neurite length were 10.0, 9.5, 0.2 and 0.9 nM and the IC_50_ for branch point formation were 8.0, 7.4, 0.2 and 0.7 nM for 4AZA2891, 4AZA2996, vincristine and vinblastine respectively. These values are in the same concentration range than the IC_50_ values measured for proliferation inhibition in cancer cell lines.

## Discussion

Microtubules play a critical role in intracellular transport, protein trafficking, and cell division, and are thus an attractive target for anti-cancer therapy. Microtubule-targeting agents alter the stability and dynamics of tubulin association and dissociation to and from microtubule ends, respectively. Available microtubule-targeting agents are central in the treatment of a wide variety of adult and pediatric cancers [1, 2]. Here, we report 3-nitropyridine analogues as a novel group of microtubule-targeting agents with potent anti-cancer effect against a broad range of cancer types. Our research reports the synthesis, biochemical and structural characterization, and *in vivo* evaluation of 3-nitro pyridine analogues as proapoptotic and anticancer compounds. 3-Nitropyridines have been described before as anti-cancer agents with CC_50_ values in the low μM range for the best derivatives [24]. The specific substitution pattern on the 3-nitropyridine scaffold of 4AZA2891 and 4AZA2996 gives rise to a 100-fold increased *in vitro* cytotoxicity. In the NCI-60 Human Tumor Cell Lines Screen, all cell lines were sensitive to the compounds and several of them with a GI_50_ below 10 nM, demonstrating a universal mechanism of action with a very high potency, excluding the 2 compounds as substrates for the classical mechanisms of resistance (PgP, MRP, etc.). *In vitro*, 3-nitropyridine compounds were selective for rapidly dividing cancer cells while they did not affect healthy cells. Their anti-cancer effect was further demonstrated *in vivo* in a murine heterotopic xenograft model of colon cancer. 4AZA2891 effectively inhibits cancer growth when administrated intravenously.

Analyses of the effect on cell cycle progression of 3-nitropyridine compounds identified them as microtubule-targeting agents. Microtubule-targeting agents promote cancer cell death by arresting the cell cycle in the G2-M phase resulting in a continued activation of the mitotic spindle assembly checkpoint and apoptosis [5, 6]. This was confirmed by microscopic study of A549 cells demonstrating a similar pattern of tubulin disintegration after treatment with the 3-nitropyridines and vincristine. Moreover, 3-nitropyridine analogues were shown to inhibit tubulin polymerization in a dose dependent manner in an *in vitro* tubulin polymerization assay. The X-ray crystallography study presented in this work establishes 4AZA2996 as a colchicine-site binding agent. The study further suggests that 4AZA2996 inhibits the curved-to-straight tubulin conformational transition with a similar mechanism as colchicine and other colchicine-site agents [4].

Pharmacokinetic studies demonstrated an elimination half-life of 0.7 hours, a volume of distribution at steady state of 5.7 l/kg. Interestingly, the compound 4AZA2891 was characterized by a preferential accumulation in the brain and kidney.

Microtubule-targeting agents are typically associated with significant hematologic and neurologic side effects. Peripheral neuropathy caused by microtubule-targeting agents is caused by microtubule damage in the long axons of peripheral nerves [9], and results in partially or non-reversible symptoms [10, 11]. These side effects often require dose reduction or discontinuation of these drugs [7, 8], and prevent the full potential of currently available microtubule-targeting agents. The 3-nitropyridine compounds described here are not associated with myelotoxicity, as demonstrated with *in vivo* experiments in Balb/c mice, but do show residual effects on neurite outgrowth in *in vitro* experiments. The observed varying toxicities can possibly be explained by varying affinity to tubulin isoforms associated with either cell type or by drug metabolism and clearance rates from the bloodstream, influencing exposure to myeloid cells.

In conclusion, we here describe 3-nitropyridine compounds as potent anti-cancer drugs against a broad range of cancer types. 3-nitropyridine analogues are novel members of the microtubule-targeting agents class as they destabilize tubulin polymerization in a dose dependent manner by interacting with tubulin in the colchicine site at the interface between the α- and β-tubulin subunits. Whereas 3-nitropyridine compounds do not induce myelotoxicity at pharmacological doses, the neurotoxicity associated to microtubule targeting agents is still present.

## Supporting information

S1 File General information. (DOCX)

S1 Fig Proton and carbon NMR spectra (**A**) ^1^H NMR of 6-chloro-N-(2-morpholinoethylamino)-3-nitropyridine. (**B**) ^13^C NMR of 6-chloro-N-(2-morpholinoethylamino)-3-nitropyridine. (**C**) ^1^H NMR of 4AZA2891. (**D**) ^13^C NMR of 4AZA2891. (**E**) ^1^H NMR of 4AZA2996. (**F**) ^13^C NMR of 4AZA2996.
(PDF)

S2 Fig Electron density maps of T_2_R-TTL-4AZA2996. (**A**) SigmaA-weighted 2mFo—DFc (dark blue mesh) and mFo—DFc (light green mesh) omit maps contoured at +1.0σ and +3.0σ, respectively. The map calculation excluded the atoms of the bound 4AZA2996 ligand. (**B**) View of the electron-density map after final refinement highlighting the ligand bound to the colchicine-site. The SigmaA-weighted 2mFo—DFc electron-density map (dark blue) is contoured at 1.0σ, the mFo-Fc map is contoured at + 3.0σ (green) and– 3.0σ (red), respectively.
(TIF)

S1 Table Data collection and refinement statistics. (DOCX)
