# Supplementary material for: 3-nitropyridine analogues as novel microtubule-targeting agents
Source: PLoS One. 2024 Nov 7;19(11):e0307153. doi: 10.1371/journal.pone.0307153 (PMC11542830; doi:10.1371/journal.pone.0307153)
Supplement: S1 File — (DOCX) [file pone.0307153.s001.docx]

**General information**

^1^H and ^13^C NMR spectra were recorded on a Bruker Avance 300 MHz instrument (^1^H NMR, 300 MHz; ^13^C NMR, 75 MHz)), using tetramethylsilane as internal standard for ^1^H NMR spectra and CDCl_3_ (77.2 ppm) for ^13^C NMR spectra and are shown in Fig S1. The spectra were processed with Bruker Topspin 2.1 software. Chemical shifts (δ) were expressed in parts per million (ppm). Abbreviations used are s = singlet, d = doublet, t = triplet, q = quartet, m = multiplet, b = broad. High resolution mass spectra were measured on a quadrupole orthogonal acceleration time-of-flight mass spectrometer (Synapt G2 HDMS, Waters, Milford, MA). Samples were infused at 3 µL/min and spectra were obtained in positive or negative ionization mode with a resolution of 15000 (FWHM) using leucine enkephalin as lock mass. Chemicals of analytical and synthetic grade were obtained from commercial sources and were used as such. Flash silica column chromatography was performed on silica gel 60 A, 0.035–0.070 mm (Acros Organics).

### **Synthesis of 6-chloro-N-(2-morpholinoethylamino)-3-nitropyridine**

To a mixture of 2, 6-chloro-3-nitropyridine (1.92 g, 10.0 mmol) and 4-(2-aminoethyl)morpholine (1.30 g, 10.0 mmol) in ethanol (20 mL) was added potassium carbonate (1.38 g, 10.0 mmol). The resulting mixture was stirred at room temperature for 4 hours. After filtration, the filtrate was concentrated under reduced pressure to yield a yellow solid. The crude product was purified by flash column chromatography on silica gel (using a mixture of ethyl acetate in dichloromethane as mobile phase, in a gradient gradually ranging from 0 to 20% ethyl acetate) to yield the title compound as a yellowish solid (2.20 g, 77%).

^1^H NMR (300 MHz, CDCl_3_): δ = 8.82 (br s, 1H), 8.35 (d, J = 8.6 Hz, 1H), 6.60 (d, J = 8.6 Hz, 1H), 3.74 (m, 6H), 2.67 (m, 2H), 2.55 (m, 4H) ppm.

^13^C NMR (75 MHz, CDCl_3_): δ = 156.8, 152.2, 137.8, 126.9, 111.7, 67.1, 56.4, 53.4, 38.0 ppm.

HRMS (ES+) calcd for C_11_H_15_ClN_4_O_3_ [M+H]+ 287.09, found 287.10.

**Synthesis of 6-(3,5-dimethylanilino)-2-(2-morpholinoethylamino)-3-nitropyridine (4AZA2891)**

A mixture of 6-chloro-2-(2-morpholinoethylamino)-3-nitropyridine (574 mg, 2.0 mmol), 3,5-dimethylaniline (726 mg, 6.0 mmol) and potassium carbonate (828 mg, 6.0 mmol) in 1,4-dioxane (10 mL) was heated under reflux for 24 hours. After cooling to room temperature, the reaction mixture was filtrated. The filtrate was concentrated under reduced pressure to yield a yellow solid. The crude product was purified by flash column chromatography on silica gel (using a mixture of methanol in dichloromethane as mobile phase, in a gradient gradually ranging from 0 to 10% methanol) to yield the title compound as a yellow solid (600 mg, 81%). Mp: 183°C.

^1^H NMR (300 MHz, CDCl_3_): δ = 9.10 (br s, 1H), 8.22 (d, J = 9.2 Hz, 1H), 7.10 (s, 2H), 6.92 (s, 1H), 6.82 (s,1H), 6.03 (d, J = 9.2 Hz, 1H), 3.73 (m, 6H), 2.66 (m, 2H), 2.51 (m, 4H), 2.32 (s, 6H) ppm.

^13^C NMR (300 MHz, CDCl_3_): δ = 158.7, 154.0, 139.0, 138.2, 136.9, 126.6, 120.6, 119.8, 99.7, 67.1, 56.9, 53.5, 38.1, 21.5 ppm.

HRMS (ES+) calcd for C_19_H_25_N_5_O_3_ [M+H]^+^ 372.2030, found 372.2032.

**Synthesis of 6-(4-methylpyridin-2-yl)-2-(2-morpholinoethylamino)-3-nitropyridine (4AZA2996)**

A mixture of 6-chloro-2-(2-morpholinoethylamino)-3-nitropyridine (574 mg, 2.0 mmol), 2-amino-4-methylpyridine (324 mg, 3.0 mmol), CsCO_3_ (1.30 g, 4.0 mmol), (±)-2,2′-bis(diphenylphosphino)-1,1′-binaphthalene (100 mg, 0.16 mmol) and tris(dibenzylideneacetone)dipalladium(0) (24 mg, 0.10 mmol) in toluene (20 mL) was degassed by vacuum and refilled with argon (3 times). The resulting mixture was heated under reflux for 4 h. After cooling to room temperature, the mixture was partitioned between dichloromethane (100 mL) and brine (20 mL). After drying over MgSO_4_, the organic phase was filtrated and concentrated to dryness. The residue was purified by flash column chromatography on silica gel (using a mixture of acetone in dichloromethane as mobile phase, in a gradient gradually ranging from 0 to 30%) to yield the title compound as a yellow solid (515 mg, 72%). Mp: 179°C.

^1^H NMR (300 MHz, CDCl_3_): δ = 9.13 (br. s, 1H), 8.45 (br., 1H), 8.28 (d, J = 9.1 Hz, 1H), 8.18 (d, J = 5.1 Hz, 1H), 8.01 (s, 1H), 6.86 (d, J = 5.1 Hz, 1H), 6.35 (d, J = 9.1 Hz, 1H), 3.76 (m, 6H), 2.71 (m, 2H), 2.54 (m, 4H), 2.40 (s, 3H) ppm.

^13^C NMR (75 MHz, CDCl_3_): δ = 157.2, 153.6, 152.6, 149.5, 147.7, 137.0, 121.4, 120.1, 114.5, 101.4, 67.1, 56.7, 53.5, 38.4, 21.7

ppm.

HRMS (ES+) calcd for C_17_H_22_N_6_O_3_ [M+H]^+^ 359.1826, found 359.1828.
