# Supplementary figures and images for: 3-nitropyridine analogues as novel microtubule-targeting agents
Source: PLoS One. 2024 Nov 7;19(11):e0307153. doi: 10.1371/journal.pone.0307153 (PMC11542830; doi:10.1371/journal.pone.0307153)

A

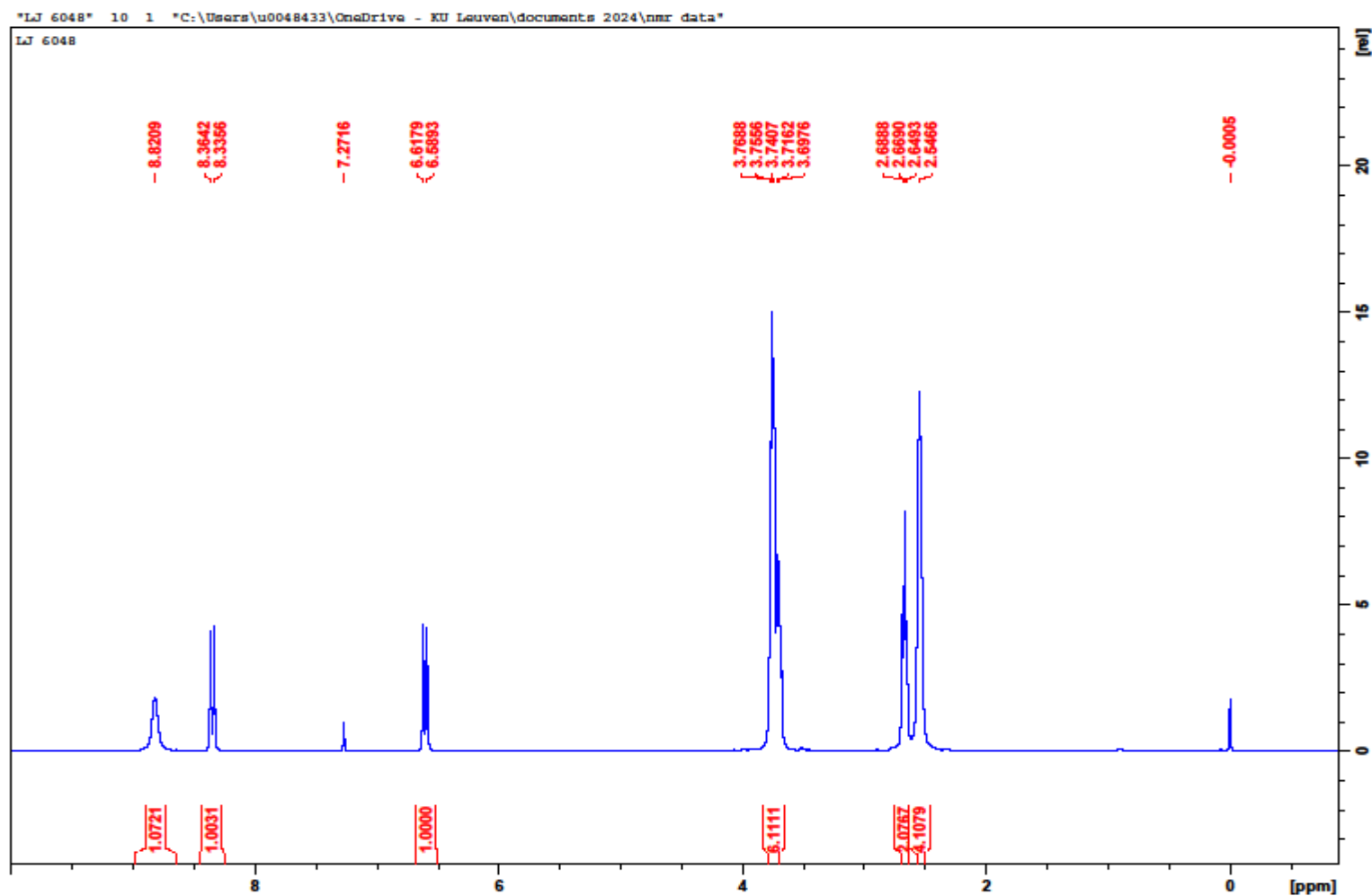

B

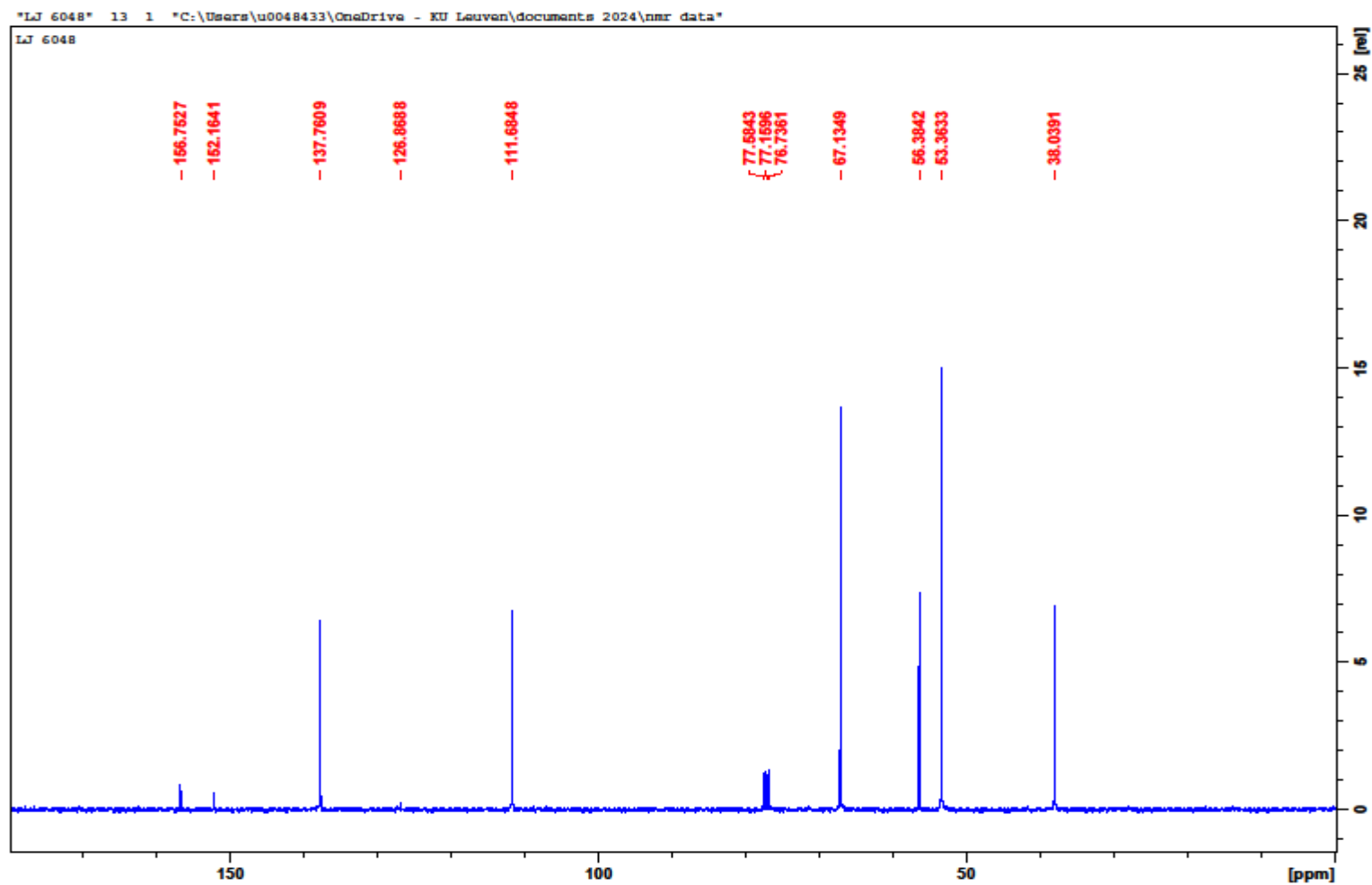

C

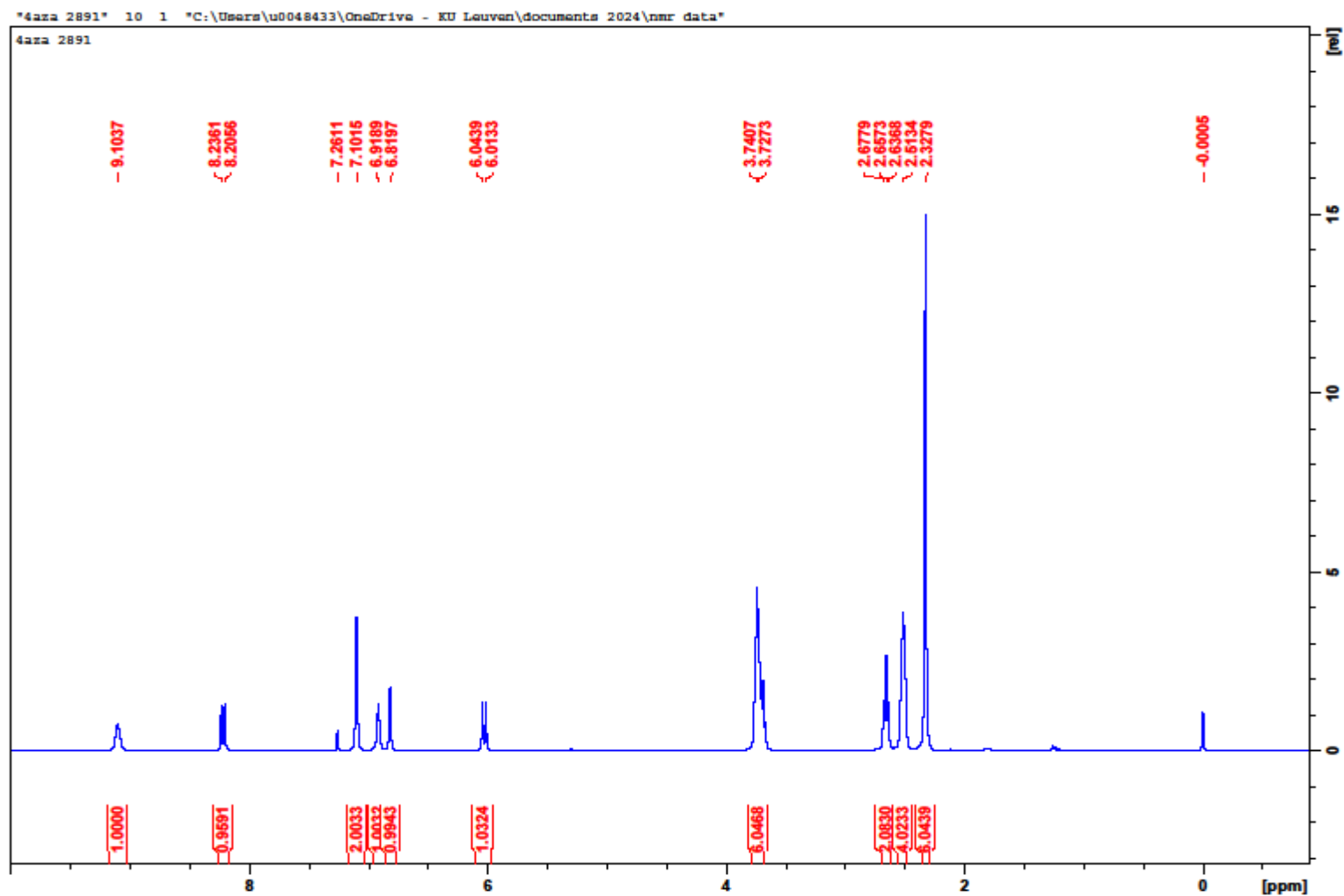

D

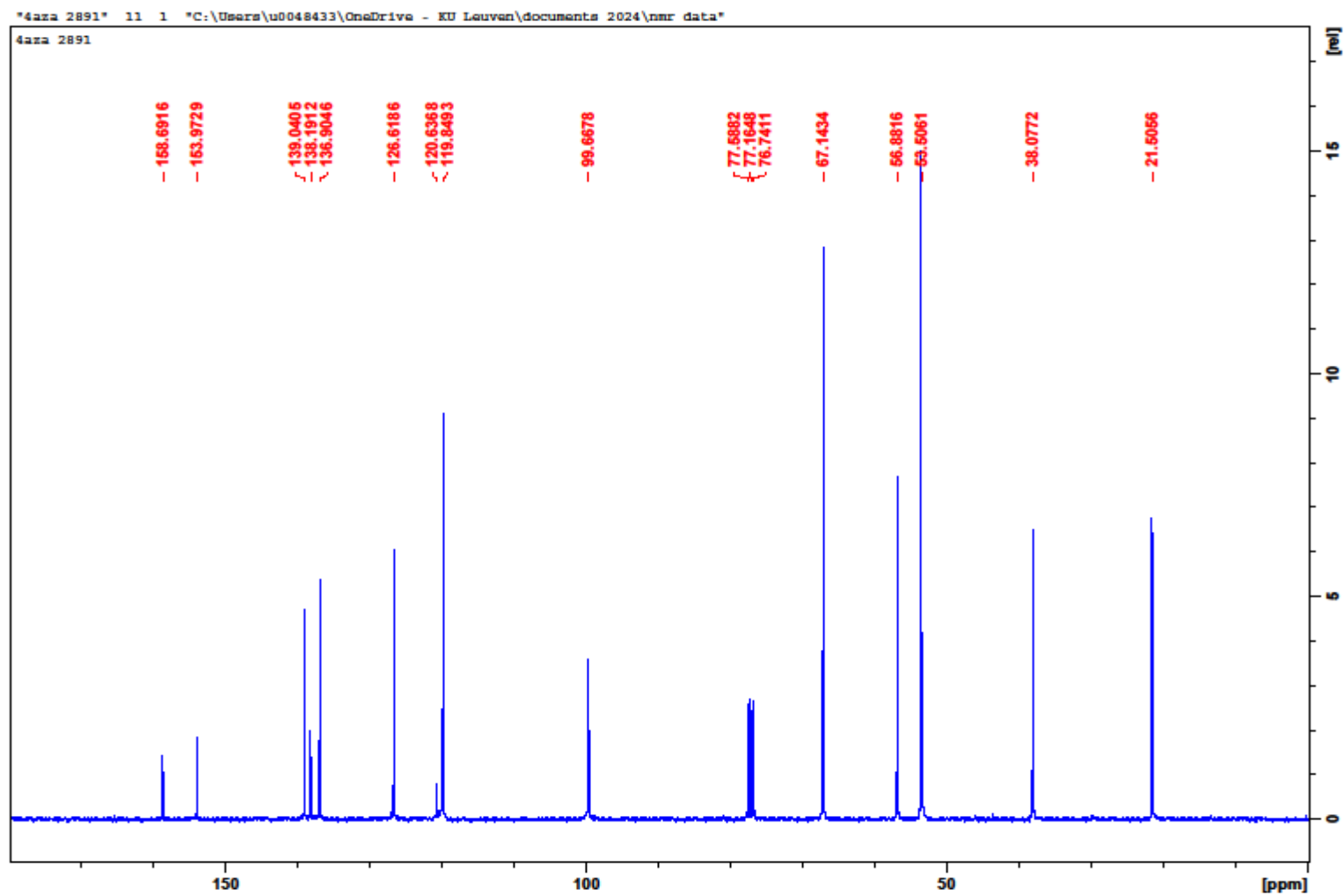

E

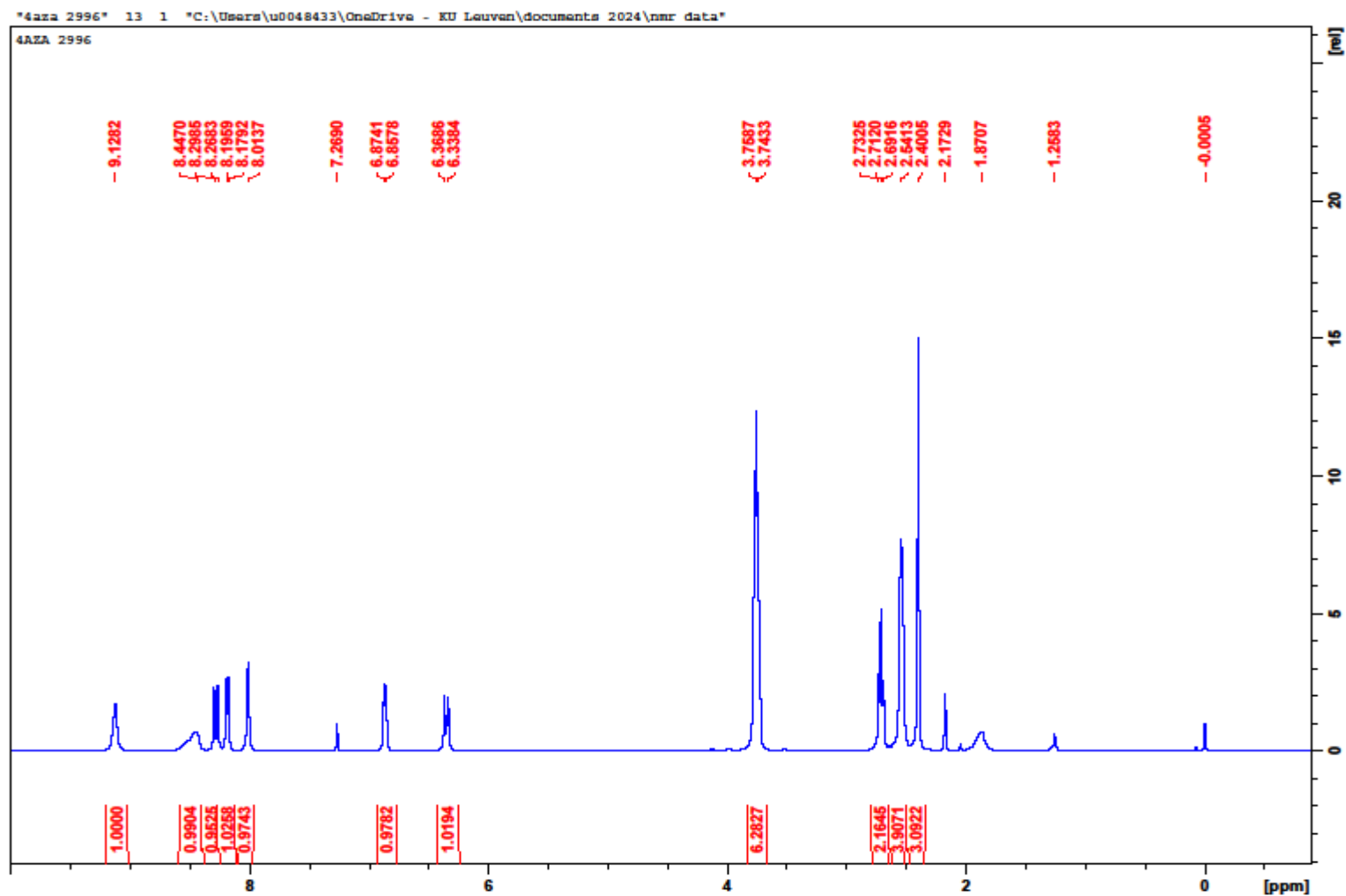

F

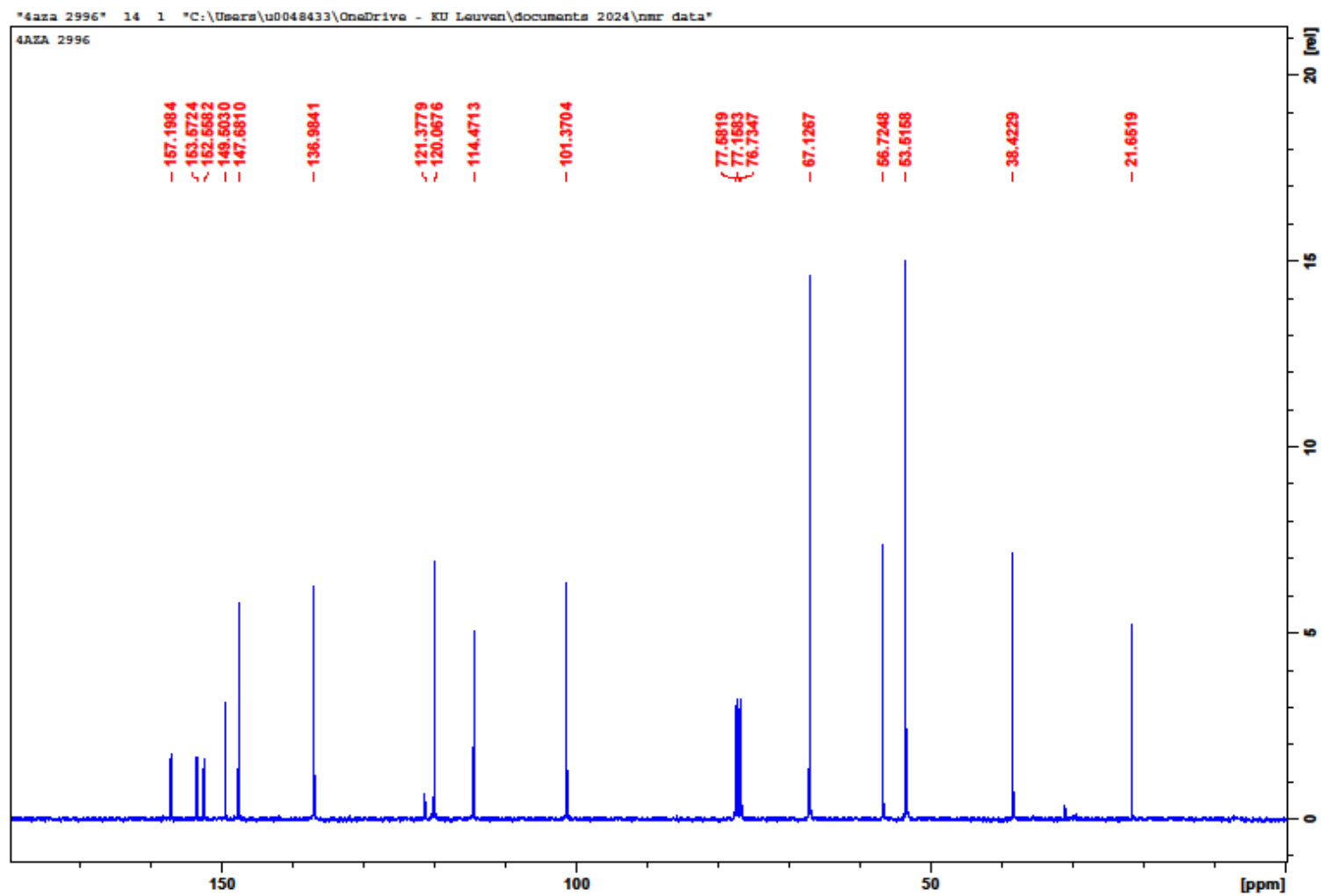

Supplement: S1 Fig — Proton and carbon NMR spectra (A) 1H NMR of 6-chloro-N-(2-morpholinoethylamino)-3-nitropyridine. (B) 13C NMR of 6-chloro-N-(2-morpholinoethylamino)-3-nitropyridine. (C) 1H NMR of 4AZA2891. (D) 13C NMR of 4AZA2891. (E) 1H NMR of 4AZA2996. (F) 13C NMR of 4AZA2996. (PDF) [file pone.0307153.s002.pdf]

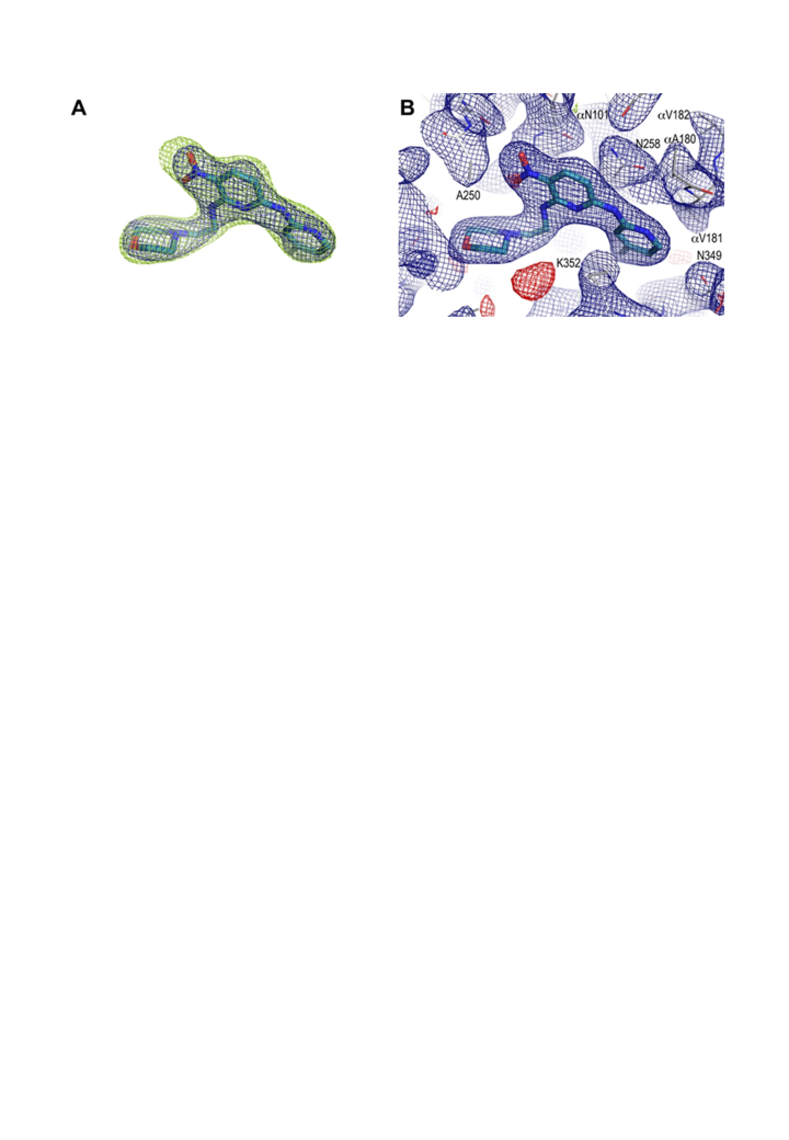

Supplement: S2 Fig — (A) SigmaA-weighted 2mFo—DFc (dark blue mesh) and mFo—DFc (light green mesh) omit maps contoured at +1.0σ and +3.0σ, respectively. The map calculation excluded the atoms of the bound 4AZA2996 ligand. (B) View of the electron-density map after final refinement highlighting the ligand bound to the colchicine-site. The SigmaA-weighted 2mFo—DFc electron-density map (dark blue) is contoured at 1.0σ, the mFo-Fc map is contoured at + 3.0σ (green) and– 3.0σ (red), respectively. (TIF) [file pone.0307153.s003.tif]
