# Supplementary material for: 3-nitropyridine analogues as novel microtubule-targeting agents
Source: PLoS One. 2024 Nov 7;19(11):e0307153. doi: 10.1371/journal.pone.0307153 (PMC11542830; doi:10.1371/journal.pone.0307153)
Supplement: S1 Table — (DOCX) [file pone.0307153.s004.docx]

|  | **T_2_R-TTL-4AZA2996** |
| --- | --- |
| **Data collection** |  |
| **Space group** | P 2_1_ 2_1_ 2_1_ |
| **Unit cell dimensions**  *a, b, c* (Å) | 105.3 158.2 182.7 |
| **Resolution range (Å)** | 48.2 - 2.2 (2.279 - 2.2) |
| **Total reflections** | 520600 (50183) |
| **Unique reflections** | 153690 (14872) |
| **Multiplicity** | 3.4 (3.4) |
| **Completeness (%)** | 99.27 (97.30) |
| **Mean I/sigma(I)** | 10.56 (0.57) |
| **Wilson B-factor** | 49.15 |
| **R_merge_** | 0.09849 (2.133) |
| **R_meas_** | 0.1171 (2.537) |
| **R_pim_** | 0.06235 (1.354) |
| **CC_1/2_** | 0.998 (0.178) |
| **CC*** | 0.999 (0.549) |
|  |  |
| **Refinement** |  |
| **Reflections used in refinement** | 153664 (14873) |
| **Reflections used for R-free** | 7683 (743) |
| **R-work** | 0.2080 (0.3767) |
| **R-free** | 0.2330 (0.3867) |
| **CC(work)** | 0.949 (0.397) |
| **CC(free)** | 0.935 (0.432) |
| **Number of non-hydrogen atoms** | 17687 |
| **macromolecules** | 17122 |
| **ligands** | 223 |
| **solvent** | 342 |
| **Protein residues** | 2166 |
| **RMS(bonds)** | 0.002 |
| **RMS(angles)** | 0.47 |
|  |  |
| **Ramachandran statistics** |  |
| **Favored regions (%)** | 97.99 |
| **Allowed (%)** | 1.96 |
| **Outliers (%)** | 0.05 |
| **Rotamer outliers (%)** | 0.96 |
| **Clashscore** | 10.34 |
| **Average B-factor** | 69.77 |
| **macromolecules** | 70.18 |
| **ligands** | 59.55 |
| **solvent** | 55.55 |
| **Number of TLS groups** | 32 |
